# Outcome measures in rheumatoid arthritis randomised trials over the last 50 years

**DOI:** 10.1186/1745-6215-14-324

**Published:** 2013-10-09

**Authors:** Jamie J Kirkham, Maarten Boers, Peter Tugwell, Mike Clarke, Paula R Williamson

**Affiliations:** 1 Department of Biostatistics, University of Liverpool, L69 3GA, Liverpool, United Kingdom; 2 Department of Epidemiology and Biostatistics, VU Medical Center, Amsterdam, the Netherlands; 3 Department of Medicine, University of Ottawa, K1N 6N5, Ottawa, Ontario, Canada; 4 Centre for Public Health, Queen’s University Belfast, BT12 6BA, Belfast, United Kingdom

**Keywords:** COMET, Core outcome set, OMERACT, Outcome reporting bias, Rheumatoid arthritis

## Abstract

**Background:**

The development and application of standardised sets of outcomes to be measured and reported in clinical trials have the potential to increase the efficiency and value of research. One of the most notable of the current outcome sets began nearly 20 years ago: the World Health Organization and International League of Associations for Rheumatology core set of outcomes for rheumatoid arthritis clinical trials, originating from the OMERACT (Outcome Measures in Rheumatology) Initiative. This study assesses the use of this core outcome set by randomised trials in rheumatology.

**Methods:**

An observational review was carried out of 350 randomised trials for the treatment of rheumatoid arthritis identified through *The Cochrane Library* (up to and including September 2012 issue). Reports of these trials were evaluated to determine whether or not there were trends in the proportion of trials reporting on the full set of core outcomes over time. Researchers who conducted trials after the publication of the core set were contacted to assess their awareness of it and to collect reasons for non-inclusion of the full core set of outcomes in the study.

**Results:**

Since the introduction of the core set of outcomes for rheumatoid arthritis, the consistency of measurement of the core set of outcomes has improved, although variation in the choice of measurement instrument remains. The majority of trialists who responded said that they would consider using the core outcome set in the design of a new trial.

**Conclusions:**

This observational review suggests that a higher percentage of trialists conducting trials in rheumatoid arthritis are now measuring the rheumatoid arthritis core outcome set. Core outcome sets have the potential to improve the evidence base for health care, but consideration must be given to the methods for disseminating their availability amongst the relevant communities.

## Background

The selection of appropriate outcome measures is crucial to the design of randomised trials. Outcomes need to be relevant to patients, practitioners and policy-makers if the findings of the research are to influence practice and future research. Poor or inappropriate selection and measurement of outcomes is not the only problem. Research has shown that outcome reporting bias, that is, results-based selection for publication of a subset of the original outcome variables, is also a major problem in randomised trials [1]. An empirical study of the impact of outcome reporting bias in randomised trials on the results of Cochrane systematic reviews found that this type of bias is an 'under-recognised problem that affects the conclusions in a substantial proportion of Cochrane reviews’ [2].

One way to mitigate the problems described above is the introduction of an agreed minimum set of standardised outcomes, to be measured and reported in all trials for a particular disease or condition, referred to as a core outcome set (COS) [3,4].

In rheumatoid arthritis (RA), it has been common practice since the 1950s to use a selection of traditional measures to define the endpoints of most clinical trials, usually including measures based on the clinician’s physical examination, global assessment, laboratory measurements and sometimes radiographs. However, during the 1980s, it became clearer to researchers that the measures chosen rarely included patient reported outcomes, were not comprehensive, some were insensitive to change, and others measured the same phenomenon and were thus redundant [5]. Furthermore, it was notable that the choice of outcomes was variable between trials: for instance, clinical trials of patients with RA in the USA measured different outcomes to those conducted in Europe [6].

Following the first OMERACT (Outcome Measures in Rheumatology) conference in 1992, and aided by meetings of experts convened by the American and European rheumatology organisations (American College of Rheumatology (ACR) and European League Against Rheumatism (EULAR)), the World Health Organization (WHO) and International League of Associations for Rheumatology (ILAR) in 1994 ratified a core set of outcomes for clinical trials of symptom-modifying anti-rheumatic drugs in RA [7]: tender joints, swollen joints, pain, physician global assessment, patient global assessment, physical disability and acute phase reactants. In trials lasting at least one year, an additional recommendation was that radiographs of the joints be taken to assess radiological damage.

In this paper we investigate whether outcome selection in RA trials has changed over time, in particular whether the RA COS is more frequently measured since the introduction of the core set of outcomes for RA and the publication of relevant regularity guidance for RA. We also question lead authors of recently published RA trials in the study cohort to gain a better understanding of trialists’ awareness of the COS and to establish reasons for non-inclusion of these outcomes in their trials.

## Methods

We searched *The Cochrane Library* to identify all Cochrane Reviews (up to and including September 2012 issue) that had considered both pharmacological and non-pharmacological interventions for the treatment of RA. Reviews were excluded if they considered only drug safety because the focus of the current study was on measures of benefit. Reviews were also excluded if no eligible randomised trials were identified, or if the review was marked as 'withdrawn’ in *The Cochrane Library* or was an overview of systematic reviews. All reports for each randomised trial included in eligible reviews were obtained for evaluation. Non-randomised trials and trials without full publications were excluded. Eligible trials that appeared in multiple reviews were evaluated once.

### Assessment of trial reports

For each review, a matrix was constructed [8] (http://ctrc.liv.ac.uk/orbit/) that listed all outcomes reported in each trial. An example outcome matrix is given in Additional file 1: Figure S1, showing whether or not trials reported on the full RA COS. For each core outcome, two members of the OMERACT executive committee (MB and PT) agreed on acceptable measurement instruments. If trial authors had used a composite outcome measure (for example, Disease Activity Score [9] or ACR improvement criteria [10]), all the individual core outcomes included in the composite were considered to have been measured, even if not reported on separately.

### Evaluation of the core outcome set

Trials were grouped according to 'topics’ as listed on *The Cochrane Library* for reviews in RA. Topics were separated into pharmacological and non pharmacological interventions. The former comprised disease-modifying anti-rheumatic drugs (DMARDs), previously also known as slow-acting anti-rheumatic drugs; symptom modifying anti-rheumatic drugs; glucocorticoids (currently classified as steroids on *The Cochrane Library*); and biologics. Non-pharmacological interventions were alternative therapies, assistive technology, diet, exercise, rehabilitation and surgery. To assess how the measurement of core outcomes had changed over time, we ordered the trials according to publication date, divided them into blocks of ten and calculated a moving average of the proportion reporting the full set. Results were presented separately for pharmacological and non-pharmacological intervention types and plotted for a period where there were a reasonable number of trials. To examine the possibility that any improvement in RA COS uptake resulted from more recent trials reporting a higher number of outcomes generally or as a result of Food and Drug Administration [11] or European Medicines Agency [12] regularity guidance for the treatment of RA, we also produce a similar moving average for the total number of clinical outcomes (core and non-core) over time and denoted the points at which important regularity guidelines for RA were first published.

For each topic, we calculated the proportion of trials that reported on the full RA COS before and after it was published. A decision to classify randomised trials published up to 1994 (the year of the RA COS publication) as 'pre-RA COS’, and the remainder as 'post-RA COS’, was made *a priori*. We present the results for the full outcome set (eight outcomes) for trials lasting a year or longer, and exclude radiological damage from the assessment (seven outcomes) for trials less than one year. Patterns in the reporting of each individual core outcome were also examined.

We also compared the measurement of the individual core clinical outcomes and laboratory measurements reported between the pre-RA COS and post-RA COS trial groups for both pharmacological and non-pharmacological interventions. For laboratory measurements, trials were classified according to whether they measured any of the following: acute phase reactants, haematology, biochemistry, urinalysis, autoimmune antibodies and specialised immunology. The frequency of reporting of the different acceptable measurement instruments used for any of the core outcomes were recorded, as were any additional non-core clinical outcomes.

### Contact with trialists

For trials that were published post-RA COS (1995 onwards), the trialists were contacted via email. Trialists were asked about their awareness of the COS during the design stage of their trial (or relevant point of outcome selection), whether or not the RA COS influenced their choice of outcomes, their awareness of the COS during final analysis and reporting, and whether or not they would consider using the COS in future trials in this area. Trialists not reporting on the full COS were also asked about reasons for not using it and the considerations that led to the outcomes that they did measure.

## Results

**Figure 1:**
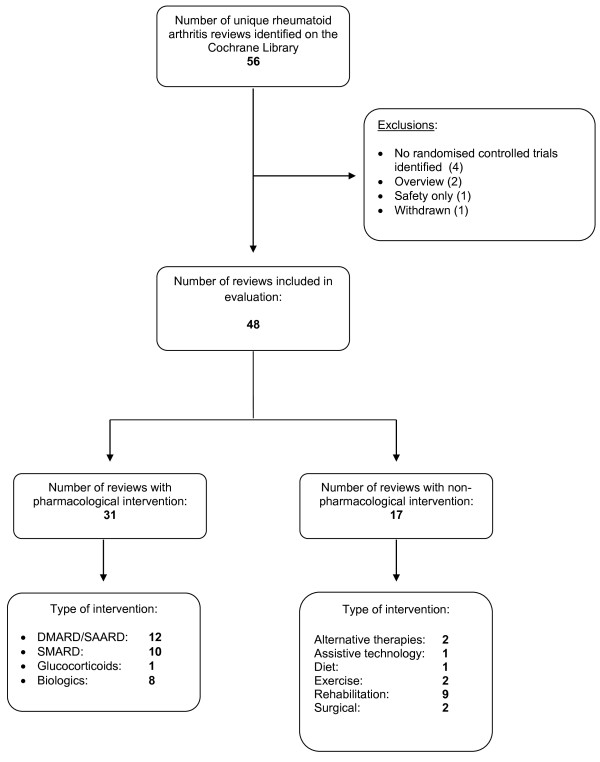
**Flow diagram of rheumatoid arthritis Cochrane systematic reviews included in study.** DMARD, disease-modifying anti-rheumatic drugs; SAARD, slow-acting anti-rheumatic drugs; SMARD, symptom-modifying anti-rheumatic drugs.

Up to and including the September 2012 issue, we found 56 eligible reviews in *The Cochrane Library*. Eight were excluded: four did not identify any randomised trials, two were overviews, one focussed on safety only and one had been withdrawn (Figure 1). Of the remaining 48 reviews, 31 focussed on a pharmacological intervention and 17 on a non-pharmacological intervention (Figure 1).

**Figure 2:**
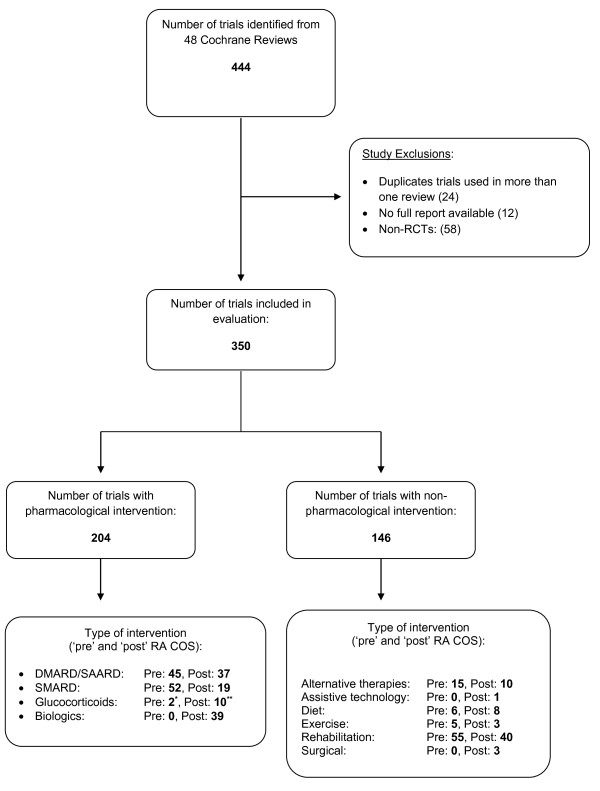
**Evaluation of randomised controlled trials within reviews.** * One duplicate trial assessed under SMARD only. ** Two duplicate trials assessed under SMARD only. SMARD, disease-modifying anti-rheumatic drugs; SAARD, slow-acting anti-rheumatic drugs; SMARD, symptom-modifying anti-rheumatic drugs.

These 48 reviews included a total of 350 randomised trials after any exclusions, 204 investigating a pharmacological intervention and 146 non-pharmacological interventions (Figure 2). About half were published post-RA COS (51% and 45% respectively; Figure 2).

### Reporting of the rheumatoid core outcome set

**Figure 3:**
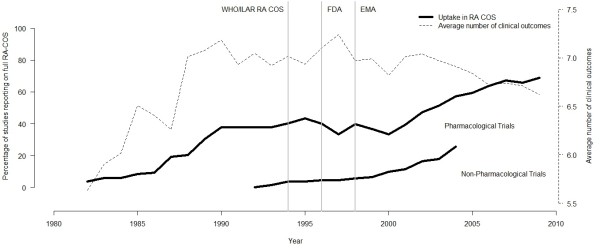
**Percentage of trials reporting on the full rheumatoid arthritis core outcome set and the average number of clinical outcomes measured over time (10-point moving yearly average).** Note: The updated European Medicines Agency guideline first came into operation in June 2004 [12], although this was based on an earlier version adopted in 1998. This guideline is currently under further revision as from 2011. The Food and Drug Administration guideline was first released in 1996 but was not formalised until 1999 [11]. There has been no formal revision of this guideline since 1999.

The reporting of the full RA COS over time is illustrated for both pharmacological and non-pharmacological intervention types in Figure 3, where a clear upward trend in the proportion of trials reporting on the full RA COS is observed. Since 1990, there does not appear to be much fluctuation in the total number of clinical outcomes measured in trials. On average most trials reported between six and seven clinical outcomes (this assessment excluded the measurement of an acute phase reactant as this was classified as a laboratory measurement). A list of acceptable core outcome measurement scales accepted in this evaluation, with frequency of use, is reported in Additional file 1: Table S1.

After the RA COS publication, marked increases were found in the measurement of the full set of RA core outcomes in pharmacologic interventions, especially DMARDs (Table 1). Despite improvement in the post-RA COS period, full RA COS reporting was generally low in symptom-modifying interventions. Biologics were introduced post-RA COS publication and most trials of these agents reported the full outcome set. There were too few trials in the glucocorticoids group to judge the impact. RA COS uptake was generally lower for trials with ≥52 weeks’ follow-up because of the additional requirement to measure radiological damage. For the pharmacological interventions, seven pharmacological trials of duration ≥52 weeks were classified as not reporting the full set because they did not measure radiological damage.

**Table 1:** Reporting of the core outcomes within the rheumatoid arthritis core outcomes set

	**Seven core outcomes (trials <52 weeks)**		**Eight core outcomes (trials ≥52 weeks)**	
**Intervention classification**	**% measured (Pre**-**RA COS)**	**% measured (Post-RA COS)**	**% measured (Pre-RA COS)**	**% measured (Post-RA COS)**
*Pharmacological*				
DMARD/SAARD	27 (9/33)	85 (17/20)	25 (3/12)	53 (9/17)
SMARD	4 (2/50)	29 (5/17)	0 (0/2)	0 (0/2)
Glucocorticoids	100 (1/1)	-	0 (0/1)	40 (4/10)
Biologics	-	93 (26/28)	-	64 (7/11)
*Non*-*pharmacological*				
Alternative therapies	15 (2/13)	88 (7/8)	0 (0/2)	0 (0/2)
Assistive technology	-	0 (0/1)	-	-
Diet	20 (1/5)	57 (4/7)	0 (0/1)	100 (1/1)
Exercise	0 (0/3)	0 (0/2)	0 (0/2)	0 (0/1)
Rehabilitation	0 (0/47)	4 (1/27)	0 (0/8)	0 (0/13)
Surgery	-	-	-	0 (0/3)

DMARD, disease-modifying anti-rheumatic drugs; pre-RA COS, randomised trials published up to 1994; post-RA COS, randomised trials published after 1994; SAARD, slow-acting anti-rheumatic drugs; SMARD, symptom-modifying anti-rheumatic drugs.

In trials of alternative therapies and dietary interventions, the measurement of the full RA COS increased post-RA COS (Table 1), however this was not observed for rehabilitation interventions. Only a few trials studied assistive technology, exercise or surgery, and none of these reported on the full RA COS.

For both pharmacological and non-pharmacological interventions, most individual outcomes were measured more frequently post-RA COS (Additional file 1: Tables S2 and S3). Tender joints, pain and physical disability were already measured frequently pre-RA COS; the largest increases occurred in swollen joint and both global measurements.

### Other outcome measures

Measurement of acute phase reactants (part of the RA COS) increased post-RA COS in trials of pharmacological and non-pharmacological interventions, whereas the number of other laboratory measurements remained similar (Additional file 1: Table S4).

In addition to the core outcomes, it was also noted that a number of trials frequently reported on a number of non-core outcomes. The most frequently reported non-core outcomes are reported in Additional file 1: Table S5.

### Contact with trialists

#### Pharmacological interventions

We were able to contact 72% (76 out of 105) of the authors from post-RA COS trials and received responses from 50% (38 out of 76), with similar response rates for those trials reporting on the full RA COS (25 out of 52, 48%) and those that did not (13 out of 24, 54%). Almost all (24 out of 25) trialists reporting the full RA COS were aware of it during the design stage. Most (21 out of 24) said that the awareness of the RA COS directly influenced their choice to measure these outcomes, and that they would also consider using the full RA COS in a new trial. Of the remaining three trialists, two felt the outcome set had been taken over by remission criteria and one was unlikely to lead a future trial eligible for this evaluation.

Most of the trialists (9 out of 13) not reporting the full RA COS were unaware of the COS during the design stage of their trial. The remaining four were aware but did not measure the full COS for the following reasons: two trials focussed mainly on safety, one trial measured but forgot to report on one of the core outcomes, and one trial did not measure physician global assessment at a time when the RA COS had been developed but not published. All 13 trialists would consider using the full RA COS in the design of a new trial.

#### Non-pharmacological interventions

We were able to contact a third (22 out of 65) of authors from the post-RA COS group of trials by email, and received responses from just four. One trial did report on the full COS (publication year 2007); the trialist was aware of the COS during the design stage of the trial and measured the RA COS as a result, and stated they would measure the full RA COS in future trials. The other three trials (publication years 1999, 2001, 2003) did not measure the full set of outcomes, with two being unaware of the RA COS during the design stage of their trial. The trialist who was aware of the RA COS did not use it as they thought it was designed for drug trials. One of the two trialists that were unaware of the COS during the design stage would consider using the RA COS in a future trial and the other trialist did not think the outcomes were all that relevant in rehabilitation trials and that the RA COS should be mainly used for assessing disease activity.

## Discussion

This study demonstrates that a community of trialists can come together to improve the consistency of outcomes that are measured. The trend over time for the number of trials reporting on the RA COS (Figure 3) was encouraging although there is still room for improvement. Despite the scope of the RA COS, there was also a suggestion that the core set of outcomes were being more frequently measured in RA trials that the RA COS was not specifically designed for, for example, non-pharmacological treatments. However, the reporting of the full RA COS for these intervention types in general remained low. The improvement in the measurement of the COS over time was not due to a general increase in the number of outcomes measured and reported in more recent trials. This includes the number of laboratory measurements, which stayed stable except for acute phase reactants (part of the COS). We acknowledge that other influential factors may also have contributed to the increase in the number of trials reporting on these core outcomes. For example, the proportion of trials reporting on the full RA COS started to rise shortly after regulatory guidelines for RA were introduced by the Food and Drug Administration and European Medicines Agency. These guidelines were, however, informed by the same organisations involved in ratifying the RA COS (EULAR, ILAR, ACR and OMERACT). Since the publication demonstrating agreement regarding the importance of these outcomes in RA trials [7], implementation of the core set has improved.

Use of the RA COS was generally lower for trials that were shorter than 52 weeks because there was an additional requirement to measure radiological damage. Responses from trialists suggest this is a resource issue, which is probably related to not only the cost of the procedure but also to the expenditure needed to obtain a valid reading of the radiographs. Some trialists also mentioned that they would prefer to report on the remission criteria as opposed to the RA COS. However, remission is still rare in RA and so important clinical effects would go unmeasured if the RA COS was not used.

The strengths of this study are that we evaluated all randomised controlled trials in all relevant Cochrane Reviews published in RA that were unselected in terms of intervention type. While we acknowledge that more trials could have been identified through a primary review of the literature, we believe that the searches carried out by Cochrane systematic reviewers are thorough and are likely to be a representative sample of all trials in RA. Before carrying out the full study, we undertook a pilot study of the 12 DMARD reviews to determine the appropriateness of the methodology and to determine acceptable outcome measures and measurement scales for each of the core outcomes. The measurement instruments that were accepted were broad; the variability in the number of methods or instruments that can be used to measure each of the core outcomes in the RA COS is a separate problem and was not addressed in this study. A summary of the types of measurement instruments that were accepted in this study and the frequency of reporting of each measurement scale is presented in Additional file 1: Table S1.

In further OMERACT work, the OMERACT Filter was implemented to select applicable measurement instruments within the chosen core set domains. As part of a patient perspective workshop at OMERACT 8 (2007), fatigue was also considered an important patient outcome in RA [13]. While this is an OMERACT recommendation, no update of the core set has yet been ratified by either of the professional organisations, ACR or EULAR. We found that only 4 of the 19 trials in our dataset published since 2007 reported on fatigue.

One potential limitation is that the assessments were carried out by one reviewer (JJK); however, the majority of the instruments used to measure outcome in RA trials are well defined (see Additional file 1: Table S1). Any uncertainties in the types of outcomes measured were referred to MB.

Barriers to the awareness of the COS still remain and this study has highlighted that the uptake of a COS can be limited or slow. For example, one trialist in this sample was unaware of the RA COS in 2001, despite its publication in 1994. It is important that the communities for which a COS is developed are aware of its existence. It was clear for many of the non-pharmacological trials that the participants included in the trial had RA (hence the RA COS was relevant), but the nature of the intervention meant that many of the primary investigators were not rheumatologists. It was encouraging to discover that 90% of trialists who responded and were likely to lead a future trial for RA would consider using the full RA COS, recognising, though, the possibility that the non-respondents might be less likely to do so. For example, trialists who had not reported on the full RA COS appeared to be more likely to respond if their trial was published closer to the 1994 publication date of the RA COS compared with those who published more recently (response rate 1995 to 2000, 57%; 2000 to 2009, 38%). This potential for response bias is important because one would expect that trials published recently were more likely to be aware of the RA COS during the design stage of the trial. Nevertheless, this wide-ranging acceptance of a minimum set of core outcomes most likely improved the consistency of research in RA, reduced heterogeneity between trials, and ensured that outcomes relevant to patient care were measured in research studies.

Placing these findings about the RA COS in a broader context, a number of other COSs have been developed across a wide range of areas of health care, examples include eczema (Harmonizing Outcome Measurements in Eczema) [14] and paediatric asthma [15]. The Core Outcome Measures in Effectiveness Trials (COMET) Initiative [16] brings together researchers interested in the development and application of COS and, as of September 2012, the COMET database contained more than 170 references to planned, on-going and completed work relevant to COSs. This report provides evidence on the successful implementation of a well-established COS in RA. Similar evaluations should be carried out for COSs developed in other therapeutic areas and the work presented here provides a template for such evaluations.

## Conclusions

The adoption of a COS has the potential to increase the consistency in outcomes measured across trials, reduce selective reporting and ensure that trials are more likely to measure appropriate outcomes. A COS for clinical trials of SMARDS in RA was first ratified in 1994 by the WHO and ILAR. This is the first study that has evaluated the reporting of a COS in trial publications and provides a template for evaluating COSs developed in other therapeutic areas.

This observational review suggests that 60% to 70% of trialists conducting trials in RA are now measuring the RA COS. Of the trialists contacted, 90% said they would consider measuring the RA COS if they were to lead a new trial in RA.

## Abbreviations

ACR: American College of Rheumatology; COS: Core outcome set; EULAR: European League Against Rheumatism; ILAR: International League of Associations for Rheumatology; OMERACT: Outcome Measures in Rheumatology; RA: Rheumatoid arthritis; WHO: World Health Organization.

## Competing interests

PT is the Co-ordinating Editor of the Cochrane Musculoskeletal Group and is a member of the OMERACT Executive Committee. MB is a member of the OMERACT Executive Committee and PRW chairs the Management Group of the COMET Initiative. MC is also a member of the COMET Management Group. JJK declares no competing interests.

## Authors’ contributions

PRW conceived the idea for the study and is the guarantor for the project. The study methods were designed by JJK, MB, PT, MC and PRW. The study articles were retrieved by JJK with the help of PT. The evaluations of the uptake of the core outcome set were carried out by JJK with the assistance of MB and PT. Email contact with trialists was done by JJK. JJK prepared the initial manuscript. MB, PT, MC and PRW were involved in the revision of this manuscript. All authors commented on and approved the final manuscript before submission. All authors read and approved the final manuscript.

## Supplementary Material

Additional file 1: Figure S1 Example of a review outcome matrix displaying the outcome information available in trial reports. **Table S1**: Accepted measurement instruments for core outcomes with frequencies of their usage across 350 intervention trials. **Table S2**: Reporting of the individual core outcomes within the RA COS (pharmacological interventions). **Table S3**: Reporting of the individual core outcomes within the RA COS (non-pharmacological interventions). **Table S4**: Reporting of laboratory measurements. **Table S5**: Non-core clinical outcomes with frequencies of their usage across 350 intervention trials. Click here for file

## References

[B1] Dwan K Altman DG Arnaiz JA Bloom J Chan AW Cronin E Decullier E Easterbrook PJ von Elm E Gamble C Ghersi D Ioannidis JPA Simes J Williamson PR Systematic review of the empirical evidence of study publication bias and outcome reporting bias PLoS One 2008 3 8 e3081 10.1371/journal.pone.0003081 18769481 PMC2518111

[B2] Kirkham JJ Dwan KM Altman DG Gamble C Dodd S Smyth R Williamson PR The impact of outcome reporting bias on systematic reviews BMJ 2010 340 c365 10.1136/bmj.c365 20156912

[B3] Clarke M Standardising outcomes for clinical trials and systematic reviews Trials 2007 8 39 10.1186/1745-6215-8-39 18039365 PMC2169261

[B4] Williamson PR Altman D Blazeby J Clarke M Gargon E Driving up the quality and relevance of research through the use of agreed core outcomes J Health Serv Res Policy 2012 17 1 1 2 10.1258/jhsrp.2011.011131 22294719

[B5] Felson DT Anderson JJ Meenan RF Time for changes in the design, analysis, and reprint of rheumatoid arthritis clinical trials Arthritis Rheum 1990 33 140 149 10.1002/art.1780330119 2405863

[B6] Tugwell P Boers M Brooks P Simon L Strande V Idzerda L OMERACT: an international initiative to improve outcome measurement in rheumatology Trials 2007 8 38 10.1186/1745-6215-8-38 18039364 PMC2169260

[B7] Boers M Tugwell P Felson DT van Riel PL Kirwan JR Edmonds JP Smolen JS Khaltaev N Muirden KD World Health Organization and International League of Associations for Rheumatology core endpoints for symptom modifying antirheumatic drugs in rheumatoid arthritis clinical trials J Rheumatol 1994 21 suppl 41 86 89 7799394

[B8] http://ctrc.liv.ac.uk/orbit/ (accessed 6 October 2013)

[B9] Van der Heijde DM Van ’t Hof MA Van Riel PL van de Putte LB Development of a disease activity score based on judgement in clinical practice by rheumatologists J Rheumatol 1993 20 579 581 8478878

[B10] Felson DT Anderson JJ Boers M Bombardier C Chernoff M Fried B Furst D Goldsmith C Kieszak S Lightfoot R Paulus H Tugwell P Weinblatt M Widmark R James Williams H Wolfe F The American college of rheumatology preliminary core set of disease activity measures for rheumatoid arthritis clinical trials. The committee on outcome measures in rheumatoid arthritis clinical trials Arthritis Rheum 1993 36 6 729 740 10.1002/art.1780360601 8507213

[B11] US Department of Health and Human Services, Food and Drug Administration Guidance for Industry Clinical Development Programs for Drugs, Devices, and Biological Products for the Treatment of Rheumatoid Arthritis (RA) 1999 [http://www.fda.gov/downloads/Drugs/GuidanceComplianceRegulatoryInformation/Guidances/UCM071579.pdf]

[B12] The European Agency for the Evaluation of Medicinal Products, Unit for the Evaluation of Medicinal Products for Human Use Guideline on Clinical Investigation of Medicinal Products other than NSAIDs for Treatment of Rheumatoid Arthritis 2003 [http://www.emea.europa.eu/docs/en_GB/document_library/Scientific_guideline/2009/09/WC500003439.pdf]

[B13] Kirwan JR Minnock P Adebajo A Bresnihan B Choy E de Wit M Hazes M Richard P Saag K Suarez-Almazor M Wells G Hewlett S Patient perspective: fatigue as a recommended patient centered outcome measure in rheumatoid arthritis J Rheumatol 2007 34 5 1174 1177 17477482

[B14] Schmitt J Langan S Stamm T Williams HC Core outcome domains for controlled trials and clinical recordkeeping in eczema: international multiperspective Delphi consensus process J Invest Dermatol 2011 131 3 623 630 10.1038/jid.2010.303 20944653

[B15] Sinha IP Gallagher R Williamson PR Smyth RL Development of a core outcome set for clinical trials in childhood asthma: a survey of clinicians, parents, and young people Trials 2012 13 103 10.1186/1745-6215-13-103 22747787 PMC3433381

[B16] COMET initiative Core Outcome Measures in Effectiveness Trials[http://www.comet-initiative.org/]

