# Supplementary material for: Outcome measures in rheumatoid arthritis randomised trials over the last 50 years
Source: Trials. 2013 Oct 9;14:324. doi: 10.1186/1745-6215-14-324 (PMC3852710; doi:10.1186/1745-6215-14-324)
Supplement: Additional file 1: Figure S1 — Example of a review outcome matrix displaying the outcome information available in trial reports. Table S1: Accepted measurement instruments for core outcomes with frequencies of their usage across 350 intervention trials. Table S2: Reporting of the individual core outcomes within the RA COS (pharmacological interventions). Table S3: Reporting of the individual core outcomes within the RA COS (non-pharmacological interventions). Table S4: Reporting of laboratory measurements. Table S5: Non-core clinical outcomes with frequencies of their usage across 350 intervention trials. [file 1745-6215-14-324-S1.docx]

**Figure S1: Example of a review outcome matrix displaying the outcome information available in trial reports**

| Study ID | Publication Date | Pre/Post  RA Core Outcome Set | Study Duration | Rheumatoid Arthritis Core Outcome Set | | | | | | | | Full Outcome Set Reported | Non-core outcomes | |
| --- | --- | --- | --- | --- | --- | --- | --- | --- | --- | --- | --- | --- | --- | --- |
|  |  |  |  | Tender joints | Swollen joints | Pain | Physician global | Patient global | Physical Disability | Acute phase reactants | Radiological damage^*^ |  | Stiffness | Quality  of Life |
| RA Study 1 | 1988 | Pre | 8 weeks | 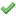 | 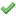 | 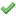 | 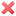 | 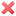 | 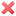 | 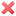 | 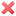 | No | 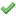 | 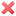 |
| RA Study 2 | 1990 | Pre | 48 weeks | 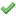 | 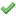 | 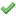 | 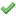 | 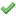 | 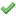 | 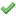 | **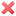** | Yes | 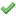 | 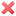 |
| RA Study 3 | 1990 | Pre | 24 weeks | 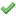 | 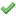 | 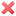 | 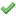 | 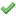 | 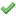 | 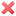 | 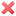 | No | 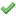 | 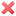 |
| RA Study 4 | 1991 | Pre | 12 weeks | 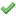 | 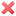 | 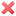 | 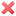 | 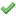 | 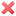 | 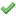 | 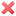 | No | 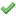 | 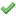 |
| RA Study 5 | 1999 | Post | 52 weeks | 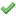 | 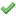 | 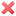 | 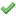 | 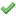 | 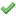 | 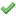 | 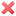 | No | 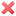 | 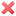 |
| RA Study 6 | 2000 | Post | 8 weeks | 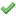 | 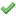 | 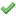 | 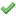 | 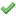 | 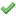 | 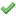 | 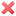 | Yes | 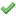 | 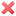 |
| RA Study 7 | 2004 | Post | 52 weeks | 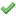 | 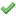 | 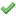 | 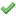 | 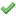 | 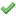 | 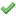 | 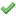 | Yes | 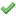 | 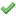 |

^*^ Outcome only required for full reporting of RA COS for trials ≥ 52 weeks.

**Table S1: Accepted measurement instruments for core outcomes with frequencies of their usage across 350 intervention trials.**

Ph: Pharmacological Interventions; N-Ph: Non-Pharmacological Interventions

| **TENDER JOINTS** | Ph | N-Ph |  | **SWOLLEN JOINTS** | Ph | N-Ph |  | **PAIN** | Ph | N-Ph |  |
| --- | --- | --- | --- | --- | --- | --- | --- | --- | --- | --- | --- |
| Ritchie Articular Index | 55 | 40 |  | Number of swollen joints | **101** | **45** |  | Pain (100mm VAS) | 110 | 79 |  |
| Modified Ritchie Articular Index | 6 | 1 |  | *20 joints* | 1 | 1 |  | Pain (3-point scale) | 4 | 0 |  |
| Articular Index (modified Landsbury) | 1 | 0 |  | *22 joints* | 1 | 0 |  | Pain (4-point scale) | 16 | 5 |  |
| Number of tender/painful joints | **96** | **40** |  | *24 joints* | 0 | 1 |  | Pain (5-point scale) | 15 | 6 |  |
| 11 joints | 0 | 1 |  | *26 joints* | 1 | 0 |  | Pain (6-point scale) | 1 | 0 |  |
| 15 joints | 0 | 1 |  | *28 joints* | 14 | 6 |  | Number of analgesics taken | 7 | 5 |  |
| 16 joints | 1 | 0 |  | *38 joints* | 1 | 0 |  | McGill Pain Questionnaire | 5 | 9 |  |
| 28 joints | 14 | 6 |  | *40 joints* | 2 | 1 |  | Modified Brief Pain Inventory | 1 | 0 |  |
| 40 joints | 2 | 0 |  | *42 joints* | 2 | 0 |  | AIMS – pain dimension | 1 | 6 |  |
| 42 joints | 1 | 0 |  | *44 joints* | 2 | 0 |  | Pain (scale not described) | 14 | 7 |  |
| 44 joints | 1 | 0 |  | *46 joints* | 4 | 1 |  | Pain Coping Inventory | 0 | 1 |  |
| 46 joints | 0 | 1 |  | *48 joints* | 1 | 0 |  |  |  |  |  |
| 48 joints | 1 | 0 |  | *49 joints* | 1 | 0 |  |  |  |  |  |
| 49 joints | 3 | 0 |  | *50 joints* | 0 | 1 |  | **PHYSICIAN GLOBAL ASSESSMENT** | Ph | N-Ph |  |
| 50 joints | 1 | 0 |  | *52 joints* | 0 | 1 |  | 3-point scale | 1 | 2 |  |
| 52 joints | 0 | 1 |  | *58 joints* | 5 | 0 |  | 4-point scale | 4 | 0 |  |
| 53 joints | 2 | 0 |  | *60 joints* | 1 | 1 |  | 5-point scale | 50 | 12 |  |
| 58 joints | 1 | 0 |  | *64 joints* | 1 | 0 |  | 7-point scale | 1 | 1 |  |
| 60 joints | 6 | 1 |  | *66 joints* | 30 | 7 |  | 11-point scale | 1 | 0 |  |
| 67 joints | 1 | 0 |  | *68 joints* | 3 | 0 |  | 100mm VAS | 49 | 8 |  |
| 68 joints | 32 | 6 |  | *74 joints* | 0 | 1 |  | Scale not specified | 10 | 6 |  |
| 71 joints | 2 | 0 |  | *Not Specified* | 31 | 24 |  |  |  |  |  |
| 77 joints | 0 | 1 |  | Joint Swelling Score | 31 | 16 |  |  |  |  |  |
| Not Specified | 28 | 22 |  | Count/score of synovial effusions | 6 | 0 |  |  |  |  |  |
| Pain / Tenderness Score | 30 | 13 |  | Number of inflamed joints | 1 | 0 |  |  |  |  |  |
| Number of active joints with synovitis | 2 | 0 |  |  |  |  |  |  |  |  |  |
| Number of inflamed joints | 1 | 0 |  |  |  |  |  |  |  |  |  |

| **PATIENT GLOBAL ASSESSMENT** | Ph | N-Ph |  | **PHYSICAL DISABILITY** | Ph | N-Ph |  | **ACUTE PHASE REACTANT** | Ph | N-Ph |
| --- | --- | --- | --- | --- | --- | --- | --- | --- | --- | --- |
| 3-point scale | 3 | 2 |  | Grip Strength | 92 | 62 |  | ESR | 144 | 66 |
| 4-point scale | 6 | 1 |  | Health Assessment Questionnaire | 75 | 38 |  | CRP | 86 | 41 |
| 5-point scale | 48 | 12 |  | Modified Health Assessment Questionnaire | 16 | 8 |  |  |  |  |
| 7-point scale | 3 | 1 |  | McMaster Toronto Arthritis Questionnaire | 2 | 2 |  | **COMPOSITE MEASURES** | Ph | N-Ph |
| 10-point scale | 1 | 0 |  | Arthritis Impact Measurement Scales (AIMS) | 3 | 23 |  | ACR 20 | 63 | 9 |
| 11-point scale | 1 | 0 |  | Steinbroker Functional Class | 14 | 0 |  | ACR 50 | 53 | 3 |
| 100mm VAS | 59 | 22 |  | SF-36 | 16 | 4 |  | ACR 70 | 46 | 2 |
| Scale not specified | 13 | 6 |  | Funktions-Fragebogen Hannover Score | 1 | 0 |  | ACR 90 | 2 | 0 |
| Modified McConkey Score | 1 | 0 |  | Disability (100mm VAS) | 1 | 1 |  | ACR-N | 5 | 0 |
| Arthritis Self-Efficacy Scale | 0 | 4 |  | Functional Capacity / Score |  |  |  | DAS 28 | 33 | 3 |
|  |  |  |  | 4-point scale | 8 | 0 |  | DAS 44 | 1 | 0 |
|  |  |  |  | 5-point scale | 4 | 0 |  | Paulus 20 | 6 | 2 |
| **RADIOLOGICAL DAMAGE** | Ph | N-Ph |  | Scale not specified | 0 | 5 |  | Paulus 50 | 3 | 1 |
| Larsen Index | 10 | 2 |  | Activities of daily living | 14 | 21 |  | DAS | 6 | 3 |
| Sharp Scoring (van der Heijde) | 19 | 1 |  | 50-ft walk time | 11 | 24 |  | Landsbury Index | 1 | 0 |
| Eroded joint count/New Erosions | 16 | 0 |  | Keitel Function Test | 3 | 1 |  | Joint count | 12 | 12 |
| Modified Sharp Score (Genant) | 1 | 0 |  | Lee Functional Index | 3 | 0 |  | Thompson Index | 2 | 2 |
| Larsen-Dale | 1 | 4 |  | Fries Index | 1 | 2 |  | Disease Activity Index (Mallya + Mace) | 1 | 0 |
| Joint Space Narrowing Score | 29 | 0 |  | Range of Motion | 0 | 23 |  | Therapeutic Remission (ACR-Pinals) | 3 | 0 |
| Erosion Score | 25 | 0 |  | General Health Questionnaire | 0 | 1 |  | EULAR Response/Remission | 15 | 0 |
| Sharp Score | 2 | 0 |  | EuroQoL | 0 | 1 |  | ACR | 4 | 0 |
| Radiograph assessment (Kellgram) | 1 | 0 |  | Hannover Functional Ability Test | 0 | 1 |  |  |  |  |
| Sharp Score (Bluhm Method) | 1 | 0 |  | Sickness Impact Profile (function) | 0 | 1 |  |  |  |  |
| Ratigen Score | 2 | 0 |  |  |  |  |  |  |  |  |
| Radiological Progression Score | 5 | 3 |  |  |  |  |  |  |  |  |
|  |  |  |  |  |  |  |  |  |  |  |

**Table S2: Reporting of the individual core outcomes within the RA COS (pharmacological interventions)**

|  | **DMARD/SAARD** | | **SMARD** | | **Glucocorticoids** | | **Biologics** |
| --- | --- | --- | --- | --- | --- | --- | --- |
|  | % Measured (Pre-RA COS) | % Measured (Post-RA COS) | % Measured (Pre-RA COS) | % Measured (Post-RA COS) | % Measured (Pre-RA COS^†^) | % Measured (Post-RA COS^‡^) | % Measured (Post-RA COS) |
| ***Core Outcome*** |  |  |  |  |  |  |  |
| Tender Joints | 98 (44/45) | 97 (36/37) | 67 (35/52) | 79 (15/19) | 100 (2/2) | 100 (10/10) | 95 (37/39) |
| Swollen Joints | 76 (34/45) | 97 (36/37) | 17 (9/52) | 63 (12/19) | 100 (2/2) | 90 (9/10) | 95 (37/39) |
| Pain | 64 (29/45) | 92 (34/37) | 87 (45/52) | 79 (15/19) | 50 (1/2) | 70 (7/10) | 97 (38/39) |
| Physician Global | 62 (28/45) | 89 (33/37) | 23 (12/52) | 58 (11/19) | 100 (2/2) | 60 (6/10) | 97 (38/39) |
| Patient Global | 71 (32/45) | 92 (34/37) | 35 (18/52) | 79 (15/19) | 50 (1/2) | 90 (9/10) | 97 (38/39) |
| Physical Disability | 89 (40/45) | 92 (34/37) | 67 (35/52) | 79 (15/19) | 100 (2/2) | 100 (10/10) | 97 (38/39) |
| Acute Phase Reactant | 93 (42/45) | 100 (37/37) | 42 (22/52) | 53 (10/19) | 100 (2/2) | 100 (10/10) | 95 (37/39) |
| Radiological Damage^*^ | 92 (11/12) | 59 (10/17) | 100 (2/2) | 100 (2/2) | 100 (1/1) | 100 (10/10) | 64 (7/11) |

^*^ Presented for trials ≥ 52 weeks in duration only.

^†^ Single duplicate trial assessed under SMARD did not report on full RA COS

^‡^ Two duplicate trials assessed under SMARD did not report on full RA COS

**Table S3: Reporting of the individual core outcomes within the RA COS (non-pharmacological interventions)**

|  | **Alternative therapies** | | **Diet** | | **Exercise** | | **Rehabilitation** | | **Surgery** |
| --- | --- | --- | --- | --- | --- | --- | --- | --- | --- |
|  | % Measured  (Pre-RA COS) | % Measured  (Post-RA COS) | % Measured  (Pre-RA COS) | % Measured  (Post-RA COS) | % Measured  (Pre-RA COS) | % Measured  (Post-RA COS) | % Measured  (Pre-RA COS) | % Measured  (Post-RA COS) | % Measured  (Post-RA COS) |
| ***Core Outcome*** |  |  |  |  |  |  |  |  |  |
| Tender Joints | 80 (12/15) | 100 (10/10) | 100 (6/6) | 100 (8/8) | 80 (4/5) | 100 (3/3) | 44 (24/55) | 55 (22/40) | 0 (0/3) |
| Swollen Joints | 53 (8/15) | 100 (10/10) | 83 (5/6) | 88 (7/8) | 100 (5/5) | 100 (3/3) | 25 (14/55) | 35 (14/40) | 0 (0/3) |
| Pain | 67 (10/15) | 100 (10/10) | 67 (4/6) | 88 (7/8) | 40 (2/5) | 33 (1/3) | 75 (41/55) | 83 (33/40) | 67 (2/3) |
| Physician Global | 33 (5/15) | 80 (8/10) | 50 (3/6) | 63 (5/8) | 0 (0/5) | 0 (0/3) | 9 (5/55) | 8 (3/40) | 0 (0/3) |
| Patient Global | 40 (6/15) | 100 (10/10) | 50 (3/6) | 88 (7/8) | 40 (2/5) | 67 (2/3) | 20 (11/55) | 25 (10/40) | 0 (0/3) |
| Physical Disability | 87 (13/15) | 90 (9/10) | 100 (6/6) | 100 (8/8) | 80 (4/5) | 100 (3/3) | 84 (46/55) | 93 (37/40) | 100 (3/3) |
| Acute Phase Reactant | 60 (9/15) | 100 (10/10) | 100 (6/6) | 100 (8/8) | 40 (2/5) | 100 (3/3) | 36 (20/55) | 28 (11/40) | 0 (0/3) |
| Radiological Damage^*^ | 0 (0/2) | 0 (0/2) | 100 (1/1) | 100 (1/1) | 50 (1/2) | 100 (1/1) | 0 (0/8) | 8 (1/13) | 67 (2/3) |

^*^ Presented for trials ≥ 52 weeks in duration only.

There was just one trial in the assistive technology group that was post-RA COS. The trial measured physical disability only.

**Table S4: Reporting of laboratory measurements**

|  | **Pharmacological interventions** | | **Non-pharmacological interventions** | |
| --- | --- | --- | --- | --- |
|  | % Measured  (Pre-RA COS) | % Measured (Post-RA COS) | % Measured  (Pre-RA COS) | % Measured (Post-RA COS) |
| ***Laboratory Outcome*** |  |  |  |  |
| Acute phase reactant^*^ | 67 (66/99) | 90 (94/105) | 68 (38/81) | 87 (32/65) |
| *Only Erythrocyte Sedimentation Rate (ESR)* | *86 (57/66)* | *18 (17/94)* | *66 (25/38)* | *13 (4/32)* |
| *Only C-reactive protein (CRP)* | *0 (0/66)* | *17 (16/94)* | *3 (1/38)* | *9 (3/32)* |
| *Both ESR/CRP* | *14 (9/66)* | *65 (61/94)* | *32 (12/38)* | *78(25/32)* |
| Haematology | 59 (58/99) | 54 (56/105) | 6 (5/81) | 25 (19/65) |
| Biochemistry | 52 (51/99) | 64 (67/105) | 19 (15/81) | 15 (10/65) |
| Urinalysis | 25 (25/99) | 26 (27/105) | 7 (6/81) | 6 (4/65) |
| Auto-immune antibodies | 34 (34/99) | 47 (49/105) | 23 (19/81) | 17 (11/65) |
| Specialised immunology | 13 (13/99) | 12 (13/105) | 10 (8/81) | 6 (4/65) |

^*^Part of the RA COS (ESR and/or CRP)

**Table S5: Non-core clinical outcomes with frequencies of their usage across 350 intervention trials.**

Outcomes were only listed if they were reported in at least two separate trials.

| **Non-Core Clinical Outcome** | **Pharmacological Interventions (n=204)** | **Non-Pharmacological Interventions (n=146)** |
| --- | --- | --- |
| \| Stiffness \| \| --- \| \| Joint ring size/circumference \| \| Fatigue \| \| Treatment preference \| \| Depression \| \| Sleep quality/tiredness \| \| Mood \| \| Joint temperature \| \| Knowledge \| \| Psychological assessment \| \| Satisfaction \| \| Self esteem \| \| Expectation \| \| Aerobic capacity/Oxygen consumption \| \| Compliance \| \| Behavioural assessment \| \| Anxiety \| \| Helplessness \| \| Coping \| \| Stress \| \| Health Locus of control \| | \| 118 \| \| --- \| \| 24 \| \| 18 \| \| 16 \| \| 9 \| \| 6 \| \| 2 \| \| 0 \| \| 0 \| \| 0 \| \| 0 \| \| 0 \| \| 0 \| \| 0 \| \| 0 \| \| 0 \| \| 0 \| \| 0 \| \| 0 \| \| 0 \| \| 0 \| | \| 67 \| \| --- \| \| 10 \| \| 8 \| \| 0 \| \| 23 \| \| 5 \| \| 0 \| \| 3 \| \| 21 \| \| 4 \| \| 13 \| \| 3 \| \| 2 \| \| 9 \| \| 10 \| \| 4 \| \| 11 \| \| 6 \| \| 8 \| \| 2 \| \| 5 \| |
